# Post-COVID-19 Pandemic Restrictions: Follow-Up on Changes Within Canadian Academic and Fieldwork Curricula

**DOI:** 10.1177/00084174241310076

**Published:** 2025-01-12

**Authors:** Diane MacKenzie, Mary Roduta Roberts, Rose Martini, Christine Ausman, Cori Schmitz

**Keywords:** Education, Entry to practice, Pandemic, Students, Universities, Enseignement, entrée en pratique, étudiants, pandémie, universités

## Abstract

**Background.** COVID-19 pandemic restrictions necessitated curricular modifications in Canadian occupational therapy education. Documentation and reflection on temporary or permanent curriculum modifications and their perceived impact on student learning and outcomes is critical. **Purpose.** To explore and compare reported curricula changes (academic and fieldwork) during restricted and post-restricted delivery periods together with the perceived impact on learners. **Method.** A cross-sectional online descriptive survey was sent to key representatives from administration, curriculum, and fieldwork at all 14 accredited occupational therapy university programs in Canada. **Findings.** Overall, many pandemic-restricted curricula delivery and assessment changes shifted back toward pre-pandemic methods. Changes that were maintained were congruent with universal design or perceived limited adverse impact on learning. Both in-person and virtual learning were perceived as important for changing practice needs. Fieldwork placement recruitment remained a challenge, with some programs increasing the use of simulation. Interpersonal competency development and assessment method integrity were more visible and of concern. **Conclusion.** Interpersonal competency development and assessment method integrity were more visible and of concern. Programs demonstrated remarkable flexibility to shift, adapt, and deliver curricula, but the human cost for this accomplishment is still palpable.

## Résumé

**Description.** Les restrictions liées à la pandémie de COVID-19 ont nécessité des modifications dans les programmes d'études canadiens en ergothérapie. La documentation et la réflexion sur les modifications temporaires ou permanentes aux programmes d'études et leur impact perçu sur l'apprentissage et les résultats des étudiants et étudiantes sont essentielles. **But.** Explorer et comparer les modifications apportées aux programmes (d'études et de stages) pendant les périodes de restrictions et après celles-ci, ainsi que l'impact perçu sur les apprenants et apprenantes. **Méthodologie.** Une enquête descriptive transversale en ligne a été envoyée aux principaux représentants de l'administration, du programme d'études et des stages des 14 programmes universitaires d'ergothérapie agréés au Canada. **Résultats.** Dans l'ensemble, de nombreux programmes d'études, restreints par la pandémie, et de nombreuses modifications des méthodes d'enseignement et d'évaluation sont revenus à des méthodes antérieures à la pandémie. Les changements qui ont été maintenus étaient conformes à la conception universelle ou ont été perçus comme ayant un impact négatif limité sur l'apprentissage. L'apprentissage en personne et l'apprentissage virtuel ont été perçus comme importants pour répondre à l'évolution des besoins de la pratique. La recherche de stages est restée un défi, certains programmes ayant accru le recours à la simulation. **Conclusion.** Le développement des compétences interpersonnelles et l'intégrité des méthodes d'évaluation étaient des aspects plus visibles et préoccupants. Les responsables ont fait preuve d'une souplesse remarquable pour modifier, adapter et offrir les programmes d'études, mais le coût humain de cette réalisation est encore perceptible.

## Introduction

The restrictive period of the COVID-19 pandemic acted as a catalyst for significant changes from in-person to online educational delivery for the global student population (UNESCO, 2021). In occupational therapy, it necessitated the rapid adoption of remote learning technologies, the development of alternative teaching and assessment methods, the revision of curricular content, and a swift response to fieldwork placement disruptions (Gill et al., 2023; MacKenzie et al., 2023). Before the pandemic, occupational therapy education was primarily delivered in person, emphasizing hands-on, experiential learning to equip students with the practical skills necessary for service provision. Curricula were structured around interactive sessions, including lab work, and fieldwork placements that facilitated the application of theoretical knowledge in real-world settings. This educational model promoted direct engagement with peers, instructors, and clients, fostering interpersonal skills crucial for therapeutic rapport-building and teamwork.

The COVID-19 pandemic forced a rapid disruption and pressured educators to innovate and adapt their academic and fieldwork curricula to maintain both the quality and continuity of occupational therapy education. The shift to remote learning raised questions about the adequacy of online platforms in conveying complex healthcare concepts and skills (Gustafsson, 2020). Synchronous online sessions attempted to replicate the immediacy of classroom interactions, while asynchronous content provided flexibility for students to manage their learning (Brown et al., 2022). However, the sudden shift raised concerns about the quality and comprehensiveness of education received by students. Additional concerns were also raised about the online learning environment contributing to social isolation, increased anxiety, decreased self-efficacy, occupational imbalance, and interruption of opportunities for developing collaborative relationships with peers, instructors, or interprofessional team members (Carolan et al., 2020; Herriott & McNulty, 2022; Kusumoto et al., 2022; Madi et al., 2023; Rasheed et al., 2020).

In the post-COVID-19 restriction era, hybrid models of education have become more commonplace, blending the advantages of both in-person and online learning, though the impact on clinical training is not yet clear (Cairney-Hill et al., 2021; Gustafsson, 2020). The main findings from a Canadian study documenting occupational therapy academic and fieldwork curricula modifications in response to pandemic restrictions (i.e., 2020–2021) included significant shifts from in-person to online delivery, resequencing or deferring of in-person programming (e.g., labs and fieldwork placements), and increased adoption of simulation and telepractice (MacKenzie et al., 2023). One of the main concerns identified during the restricted programming period was the development of interpersonal or ‘soft skills’ among students (e.g., communication and relational skills). It is important to document how occupational therapy programs have evolved to deliver their curriculum in an environment free from mandatory requirements of social distancing and remote engagement and explore whether concerns identified during the pandemic period remain.

The purpose of this follow-up study was to document Canadian occupational therapy curricula changes (both academic and fieldwork) reported in the post-restricted delivery time points for the academic years of 2021–2022 and 2022–2023. Elements examined academic instruction and assessment methods, fieldwork revisions, and the respondents’ perceived impact on student learning and outcomes.

## Method

This follow-up study planned to employ a convergent mixed methods design (Creswell & Clark, 2018) to document the Canadian occupational therapy program curricula changes as COVID-19 restrictions were removed. A cross-sectional descriptive survey with open-ended (qualitative) and closed-ended questions (quantitative) gathered information on curricula structure and delivery, teaching and assessment methodologies, fieldwork placements, and the respondent's perception of curricular adaptation's impact on learners. Survey respondents were invited to participate in a post-survey focus group or interview to enhance the rigor of the mixed method survey analysis and confirm or challenge the content validity of the survey analysis (Birt et al., 2016). This study received ethics approval from the authors’ respective university research ethics boards (Dalhousie University 2021-5446, the University of Ottawa H-04-21-6856, and the University of Alberta Pro00109656).

### Participants

Similar to the study completed during pandemic restrictions (MacKenzie et al., 2023), participants were eligible to be recruited from administration, curriculum, and fieldwork roles from all 14 Canadian occupational therapy university programs. Specific roles were targeted given their responsibilities and congruence with the information being sought regarding academic curricula, fieldwork placements, and overall program changes throughout the pandemic restriction and post-restriction period. Recruitment emails were sent to the Association of Canadian Occupational Therapy University Program (ACOTUP) directors, curriculum chairs from the Academic Education Committee (AEC), and fieldwork coordinators from the Committee on University Fieldwork Education (CUFE) with instructions to share the email with the current person(s) occupying the role.

### Procedure

The total design method (Dillman et al., 2014) informed the recruitment strategy and online follow-up survey hosted on Opinio™. The survey deployed included closed and open-ended questions. Respondents were asked about admission cohort size and completion, academic curriculum and delivery methods, and core fieldwork content during the restriction-free academic years 2021–2022 and 2022–2023. The survey was released in May 2023, two reminder emails were sent in June, and the survey closed at the end of June 2023. Data collection occurred with no in-person restrictions and Canada had established COVID-19 was no longer considered a public health emergency of international concern (Public Health Agency of Canada, 2023).

The Opinio™ online survey link was emailed to the targeted participants. Survey content included branching sections aligned with specific positions of: Program Director/Coordinator (cohort size, length of program, resources), Curriculum Chair (academic curriculum, teaching and evaluation); and Fieldwork Coordinator (fieldwork education). Recognizing participants may hold more than one position, combined role response options included: Program Director/Curriculum Chair, and Program Director/Curriculum Chair/Fieldwork Coordinator. Participants were not required to answer all the questions in their respective sections and could exit the survey at any point.

All recruitment and research tools were translated into French by a professional translation service and verified by a bilingual francophone occupational therapist. Participants could complete the survey in English or French. For ease of quantifying and reporting changes reported across varied curricular structures, and time periods the customized Likert-type scale developed from our previous study remained for the re-deployed questions (MacKenzie et al., 2023). Program representative participants were asked to report “the respective percentage of your overall curriculum (not per class) pre-COVID, during COVID, and what percentage might stay post-COVID-19” for teaching and assessment methods. The scale's anchored percentage categories (0; 25% or less; 26%–50%; 51%–75%; more than 75%) allowed for consistent descriptive reporting, and comparison with previously reported survey results. Participants were also asked to rate and provide free text responses about their “perceived impact on learners and their attainment of competencies due to changes in teaching and assessment methods.” There was no request for the evidence that informed the respondents’ ratings.

Survey participants were invited to engage in a post-survey interview or focus group by clicking on a link that took them to a separate Opinio™ site where they provided their name and email contact.

### Data Analysis

The survey results were downloaded from Opinio™ into an Excel spreadsheet (Microsoft, 2022), cleaned, and blinded by the research assistant prior to analysis by the team. The French free-text survey responses were translated using DeepL Translator (n.d.) and verified by a bilingual francophone research team member. All survey data is anonymous and reported at the aggregate level. The quantitative account of curriculum adaptations and program perceptions about the impact of COVID-19 on learner competency is reported by the frequency of program responses on teaching, assessment, fieldwork practices, and perceived impact on competencies. In some cases, not all programs responded to a question. Given the potential for overlapping roles or knowledge of respondents, if a question received conflicting responses from the same institution, the primary role for the content of the survey branch was selected.

To provide meaningful context for the categorical quantitative survey questions, qualitative description with content analysis was completed for the 13 open-ended free-text responses (Doyle et al., 2020; Elo & Kyngäs, 2008). The qualitative responses from the individual interview were reviewed using the same survey free text codes and integrated into the survey free text response analysis. Given this was a follow-up survey, a priori survey topics were used to code the responses as they appeared for each survey question. Methodological rigor and trustworthiness during data analysis and interpretation was accomplished by one author first confirming the codes which was then followed by an independent review by two other investigators. The research team discussed and resolved any discrepancies. To refine coding and detect patterns and relationships among categories, the team used a constant comparative approach. Consistencies between quantitative ratings and qualitative interpretations were compared to confirm or refute discrepancies between ratings and textual comments. The team members reviewed the interpreted findings for accuracy and completeness. During the qualitative analysis, quotes to illustrate content were identified.

### Findings

Responses were received from all 14 Canadian programs, though not all programs provided responses to all questions. Overall, 22 respondents completed the survey within the following reporting position(s): program director (one), curriculum chair (seven), fieldwork coordinator (nine), program director/curriculum chair (two), curriculum chair/fieldwork coordinator (two) and program director/curriculum/fieldwork coordinator (one) from entry to practice master's programs, including Québec programs that possess an integrated bachelor's degree. Only two respondents left their information for a follow-up interview and only one participated in the individual interview with the research assistant. The interview responses have been integrated into the free text cross-sectional analysis. Given there was only one participant in the qualitative interview, the findings in this study primarily report on the descriptive cross-sectional survey responses.

### Curricula Sequencing

Of the programs reporting on changes to their admission cohort size, three programs reported no change to admissions in 2020, 2021, and 2023. Two reported no change in 2022, one program reported a decrease in students in 2020, and 2022. Two reported a decrease in student admissions in 2021. The survey also inquired if students in the admission cohorts completed their program at the typical end date, or if their program completion was delayed. Of the 11 programs that responded regarding program completion, a typical end date was reported for seven programs in 2020, 10 in 2021, and 11 in 2022 and 2023. Four programs reported delayed program completion in 2021, one in 2022, and none in 2023. Program delays were related to difficulty obtaining sufficient fieldwork recruitment and/or fieldwork placement cancellations.

The admission cohort years from 2020 to 2023 experienced shifts in their program sequence (academic or fieldwork) due to COVID-19: five programs reported a delay in the 2020–2021 school year, six in the 2021–2022 school year, three in 2022–2023 school year, and one anticipated in 2023–2024 school year. To support students moving forward in their respective curricula, programs reported shifting academic courses to accommodate fieldwork changes, adaptations and/or limited offers, university guidelines, and public health guidelines with the transition from online to in-person formats as well as prescribed room space (e.g., when physical distancing was required, limiting occupancy). Two programs reported that no change in full course sequences ever occurred, two programs reported full courses moved earlier in a semester or year, three reported moving full courses to later in a semester or year, and five programs reported that COVID-19 changes in 2021–2022 reverted back to pre-COVID-19 curriculum in 2022–2023. Re-sequencings of the courses were necessary due to sanctioned face-to-face restrictions, with one program noting “we had an amazing leader of the curriculum shuffling.” For the majority of occupational therapy programs, the post-COVID-19 (2022–2023) trend was towards “*business as usual*” with no changes to the number of students coming in per year, and admission cohorts graduating in 2022 and 2023 within expected timelines.

### Teaching Methods and Perceived Impact on Competency Attainment

Table 1 presents the compilation and comparison of actual teaching methodologies reported in use and the perceived impact of the teaching method on competency attainment for the academic years 2021–2022 and 2022–2023.

**Table 1:** Numbers of Programs Reporting Changes in Teaching Methodology and Perceived Impact on Competency Attainment

		Number of programs reporting curriculum change^a,b^					Competency attainment^a,b^	
Teaching methodology	Time period	0%	25% or less	26%–50%	51%–75%	75% plus	No impact	Impact
F2F lecture	2021–2022		4	1		1	3	4
	2022–2023		1		1	5	3	6
Online lecture synchronous	2021–2022			6		1	3	6
	2022–2023		6			1	2	7
Online lecture asynchronous	2021–2022		4	3			3	5
	2022–2023		7				2	8
F2F labs	2021–2022		2		1	4	3	6
	2022–2023		1			6	4	5
Online lab synchronous	2021–2022	1	5	1			2	6
	2022–2023	3	4				1	4
Online lab Asynchronous	2021–2022	4	2				2	3
	2022–2023	4	2	2	1	1	1	5
F2F PBL	2021–2022	1	2	2	1	1	3	2
	2022–2023		2		1	4	5	3
Online PBL	2021–2022		3	2		2	3	4
	2022–2023		5	1		1	2	5

^a^ The percentage category reported is reflective of the overall curriculum, not specific courses.

^b^ There are two different survey questions reflected in this table which results in different program totals reporting curriculum changes and perceived impact respectively.

Teaching methods in the academic years 2021–2022 and 2022–2023 show programs continued to adopt some online instructional adaptations and innovations in a post-pandemic restricted curriculum. From 2021–2022 to 2022–2023, the return to in-person delivery increased, as noted for F2F (face-to-face) lectures, F2F labs, and F2F PBL (problem-based learning). Some schools reported retaining online synchronous labs, and compared to 2021–2022, all schools report using some form of online asynchronous labs. Challenges that arose during pandemic adjustments remained, particularly around the type of course content that could effectively be delivered online and the content that should be delivered in person. For example, delivery of kinesthetic-based learning (e.g., client transfers) remained in person for all programs as respondents reported that an online delivery method “limited hands-on skills development.” Furthermore, concern was expressed regarding “Communication skills—we feel that perhaps interpersonal communication skills have been negatively impacted by increased online learning and increased student anxiety.”

The use of recording and online access continued in some programs to allow for “enhanced participation of practice or community partners and guest lecturers joining from distance.” The use of online teaching methods born out of necessity during the restricted in-person delivery methods brought enhanced discussion of universal design for learning (UDL). In some cases, “faculty continued to provide live streaming of content for enhanced accessibility for those unable to attend in person.”

All programs (*n* = 7) reported continued practice skill delivery in a F2F format (as during pandemic restrictions) for seven practice skills in 2022–2023 (i.e., transfers, seating assessment, wheelchair and mobility skills, range of motion (ROM), manual muscle testing (MMT), sensation, splinting/orthotics, activities of daily living (ADL), and instrumental activities of daily living (IADL) training). Of the seven programs reporting, two programs changed the delivery format for five practice skills, particularly those requiring communication ability (i.e., giving and receiving feedback, interpersonal communication, group facilitation/dynamics, and adaptive equipment/technology). Some programs moved from F2F to a combination of F2F and online delivery, depending on the practice skill. For instance, one program reported retaining some online, combined with F2F for competencies around interpersonal skills to the practice skills transferrable to a telerehab setting. Combining online with F2F was also used by two programs for providing feedback, supervised practice, and adaptive equipment. For one program, group facilitation and dynamics moved entirely online. Some programs justified the maintenance of online formats to develop virtual practice competency development.

### Assessment Methods and Perceived Impact on Competency Attainment

Table 2 illustrates the reported assessment methods employed in the 2021–2022 and 2022–2023 academic years together with the perception of competency attainment. Assessments using online methods decreased from the reported use in 2021–2022, but were still “sometimes used to complement in-person assessment methods.” Relative to 2021–2022, there was an additional increase in in-person evaluation with F2F written exams. Online written exams continued in some programs with methods in place for exam integrity, while others discontinued them. A respondent expressed “concern regarding professional behavior (cheating) with online exams, students are split due to technology failure fears, less certain whether competencies are being achieved due to potential breaches in academic integrity.” Another respondent noted that their trial of using online exam integrity tools was “met with mixed success.”

**Table 2:** Numbers of Programs Reporting Changes in Assessment Methodology and Perceived Impact on Competency Attainment

		Number of programs reporting curriculum changes^a,b^					Competency attainment^a,b^	
Assessment methodology	Time period	0%	25% or less	26%–50%	51%–75%	75% plus	No impact	Impact
F2F written exams	2021–2022	1	2	3		1	5	2
	2022–2023		2	1		5	6	2
Online written exams	2021–2022		4	2		1	2	7
	2022–2023	2	5	1			4	3
Online individual oral exams	2021–2022	1	3	2	1		6	1
	2022–2023	3	3	1			3	3
Online synchronous presentations	2021–2022	2	3	1		1	5	3
	2022–2023	4	2	1		1	2	6
Online asynchronous presentations	2021–2022	1	4	2			4	1
	2022–2023	3	4				3	3
F2F OSCE with SP	2021–2022	3	4				3	3
	2022–2023	4	3				4	4
F2F OSCE with Peers/Instructors	2021–2022		4	1		2	3	3
	2022–2023	1	2	1		3	4	1
Online OSCE with SP	2021–2022	2	3			2	2	2
	2022–2023	2	2			3	2	2
Online OSCE with peers/instructors	2021–2022	5	2				2	3
	2022–2023	5	2				2	1
Written papers	2021–2022	6	1				8	0
	2022–2023	6	1				8	0

^a^ The percentage category reported is reflective of the overall curriculum, not specific courses.

^b^ There are two different survey questions reflected in this table which results in different program totals reporting curriculum changes and perceived impact respectively.

Of the seven programs reporting the use of F2F OSCEs with simulated patients (SPs), little change was noted with four programs using this assessment method in 2021–2022 compared to three programs in 2022–2023, presumably because OSCEs had to take place F2F in the first place. A small change in the use of F2F OSCEs with instructors and peers was noted. In 2021–2022, four out of eight programs used this method <25% of the program and three out of eight programs <75%. In 2022–2023, one program discontinued the method, two programs used it <25%, and four programs >75%.

The number of programs that used the online OSCEs with SP assessment method in 2021–2022 was the same in 2022–2023 (five out of eight programs reporting), with an increase noted from two to three programs employing the method >75%. Upon returning to F2F patient simulations and OSCEs, one respondent noted that … “Students tend to be more anxious and there seems to be less attention to detail around the patient context (e.g., checking surroundings before effecting a transfer).” The concern reported related to understanding how online learning impacted knowledge transfer to physical skill development (i.e., screen presentation to real environment) noted, “We are discontinuing the majority of the [online] assessment methods adopted during COVID-19.”

Three programs also reported concern regarding anxiety and evaluation methods, conjecturing that this inability to manage anxiety may also impact the attainment of competencies. Several examples were provided where student-reported anxiety was more frequent, regardless of the different evaluation formats provided (e.g., students were anxious during in-person exams in the presence of other students, students were anxious about technology failures with online exams).

### Fieldwork

The survey investigated the variety of fieldwork options programs have used since November 2021. The types of fieldwork options per learning level are displayed in Table 3.

**Table 3:** Numbers of Programs Reporting Fieldwork Placements Types Per Level November 2021–June 2023

		Number of programs Reporting curriculum changes^a,b^				
Fieldwork format	Level^b^	not applicable^c^ not offered^d^	Less than 25%	26–50%	51–75%	More than 75%
Clinical—in person	Level 1		3		3	7
	Level 2		1		2	10
	Level 3		1		4	8
Clinical—telerehab/virtual	Level 1	3	7	1		
	Level 2	3	8			
	Level 3	3	8			
Clinical—telerehab/in-person	Level 1	4	6	1		1
	Level 2	3	8	1		
	Level 3	3	8	1		
Simulation	Level 1	9				1
	Level 2	11				
	Level 3	11				
Leadership/admin/non-clinical	Level 1	7	4			
	Level 2	2	9			
	Level 3	2	10			
Role expanding/enhancing	Level 1	8	2	1		
	Level 2	3	7			
	Level 3	3	8	2		
Role emerging	Level 1	5	5	1		
	Level 2		11	1		
	Level 3		11	1		

^a^ Not all programs responded to all questions so totals per methodology may not equal 14.

^b^ The number and length of each level of fieldwork placements to achieve the minimum 1,000 h vary across Canadian occupational therapy university programs.

^c^ Not applicable (the type of placement is available but not at the level listed).

^d^ Not offered (the level of placement is not offered) data were combined for reporting purposes.

Most fieldwork placements were clinical in-person with increased offerings of Leadership, Emerging/Enhancing, Advocacy, Program Planning and Evaluation (LEAP), and role-enhancing/emerging in later fieldwork placements. Five programs (of 10 responding) reported that they were not limited in placement offers or the timing of fieldwork completion in the post-restricted period, so no new strategies were required during this period. One program used simulations for early Level 1 fieldwork placements to attain the 1,000 needed fieldwork hours and reported positive outcomes.

We used carefully-crafted simulation activities informed by best-practice simulation guidelines from WFOT partner institutions in Australia and New Zealand. The simulations were designed and delivered in partnership with the practice community to reflect the realities of practice and to further student learning with respect to key CBFE-OT competencies—particularly those competencies which had typically been challenging for students entering their first fulltime placement (e.g., facilitating change with a practice process and clinical reasoning).

Simulations were reported for Level 1 fieldwork placements only. Due to continued challenges with obtaining enough placement offerings, one program reported plans to change from F2F to simulations for Level 1 placements. Fully online placements were reported in Level 1 more so than later placements, with an acknowledgment that the use of fully virtual placements needs to be balanced with in-person clinical experiences. Reported adjustments to fieldwork placements later in the program were the result of changes made during the respective cohort's first year when pandemic restrictions created delays.

Preceptor burnout and workforce shortages were reported across Canada, particularly linked to fewer placement offers mainly for early Levels 1 and 2 fieldwork placements. The overwhelming concern reported by the programs related to *the* “reduced capacity” of fieldwork placements “and the impact COVID-19 has had on the workforce (burn-out, vacant positions, OTs switching careers) which means we are working harder than ever to recruit good quality placements for our students.” Increased demands placed upon preceptors at their worksite were cited as the reason for some placement sites having a “lower tolerance for students who may need additional support during fieldwork”. More preceptors offered senior-level placements, with fewer preceptors electing to supervise students earlier in their educational training or those having individualized trajectories. “We perceive that recruitment of lower-level placements has become more difficult. Few offers due to supervisors’ fatigue and just general staffing issues in in-health care.”

When asked about factors still impacting fieldwork, one program reported to have “ended fieldwork placements early due to the increased needs of students and demands on the preceptor”. In contrast, another program reported, that some “…sites/CE [clinical educators] have become accustomed to prioritizing Level 3 placements needs (and post-pandemic are focused on supervising with the intent/hope to hire (i.e., recruiting strategy)”. These situations created an increased workload for the fieldwork coordinators with a “greater amount of time soliciting offers for fieldwork”. The difficult reality for fieldwork placements is that “human resources shortages are significantly impacting organizations ability to offer placements”. “… our offer numbers have not “re-bounded” to pre-COVID-19 levels and continue to struggle with securing enough offers.”

### Obstacles Encountered

Many obstacles to sustaining curriculum and fieldwork delivery were reported. These obstacles tended to be around the available resources to meet the curriculum delivery environment or around the human cost to ensure programming met expected competencies. Even after post-pandemic restrictions, respondents still made references to the rapid shifts that were required to move to an online environment during 2020–2021 and the continued challenges to sustaining online and/or hybrid options. Despite the lifting of restrictions and return to F2F, the demand for hybrid delivery remained with no increase in support. The “mental load” for instructors to adopt new technologies during COVID-19 and then shift back to in-person, often came with an expectation to maintain a hybrid delivery which “challenged educators to consider new learning environments and approaches”. The following examples from three different respondents provide insight into the demands on faculty, “We did not have technical resources needed to deliver a virtual program effectively. We increased faculty resources as we needed to deliver in-person content to smaller cohorts offered multiple times”, “Increased time demand on administrative personnel and professors due to expectation that courses can be held F2F and online (managing student requests) with the lack of technology in the classroom to be able to support bimodal learning”, and “Increased requirement of instructors within each course, increase in learning curve for online resources, increased requirement for technical support (e.g., developing videos, monitoring chat during class, etc.). Increased admin support (e.g., remote learning assistants, TAs).”

Faculty raised concerns regarding the ability to meet learning outcomes with shifting delivery methods. This was particularly noted with the shift of some fieldwork hours. Finally, even though this survey inquired about the academic years 2021–2022 and 2022–2023, there were several comments regarding the ongoing human cost due to earlier curricular changes. There is clearly ongoing concern regarding the human interaction components ranging from “The abrupt change from in-person to online was a challenging transition for faculty, staff, and students. This took additional time for faculty to prepare delivery of courses and an emotional toll on faculty and students. Students felt isolated from their peers and faculty.”

The isolation also raised concerns about the blurring of lines with “Psychosocial factors were challenged due to our personal/professional lives becoming entwined during remote teaching/work.” and concerns that “fewer points of contact with students may contribute to less feedback related to professional behaviours”

### Innovative Ways to Foster a Sense of Community

Several online innovations that were reported to foster a sense of community continued even after restrictions were lifted. Continued innovations included the use of “Remote office hours, and remote accommodation meetings”; “more town halls or scheduled communication meetings” and “Frequent class meetings via Zoom with Director, entry-level program chair and fieldwork coordinator to collect student feedback”. One program experienced continued positive online interaction to purposefully address the loss of spontaneous fieldwork debriefing interactions.

Student leaders took the initiative to collaborate with the fieldwork team to host/support elective fieldwork debriefs following sessions (our team would not have had time to set this up but were grateful that the students did and it seemed well received) to replace the ‘buzz’ and opportunities that would have normally naturally occurred upon return to campus following fieldwork placements.

However, in the post-pandemic environment, other programs found that online strategies were met with limited success.

We held virtual town halls, hosted open Zoom links for students to connect and interact socially and had virtual game nights, but this aspect of building community was much harder virtually. People did not necessarily want to be on zoom after learning on Zoom all day. They needed to disconnect from technology.

Another respondent noted, “…during one session we tried organized/hosted an elective collective Zoom meeting for any students who were completing a rural placement and perhaps feeling isolated (not engaged in as anticipated so not continued)”. There was also a purposeful shift to in-person opportunities, with an emphasis on relational learning opportunities to build a sense of community with students, faculty, and the practice community. This is reflected in the following quotes made by different respondents:

We have gone back to fully in-person learning and openly discuss the challenges of remote learning, the lack of connection and the importance of relational learning.

We are hypothesizing that our students are less engaged in the class when they are online, and lose out on relational learning (i.e., engaging with peers, classroom discussion, etc.).

We are liaising with student associations to encourage in person events, recognizing the importance of the in person social interactions.

Finally, there was an emphasis on promoting wellness for students by faculty and student leaders. “Wellness sessions guided by a member of the OT program were put in place at each semester (before exams/assignment submission period).” Another respondent noted

Wellness sessions were put in place for students (led by a member of the OT program) each semester, just before students entered the period of the semester where they have multiple assignments due and exams to write. This was done to help students manage their level of anxiety and provide them with tools to manage their time, work, anxiety.

## Discussion

Occupational therapy education underwent a significant transformation in the wake of the COVID-19 pandemic. This follow-up study documents the changes reported in the post-restricted COVID-19 period from 2021–2023 in academic and fieldwork curricula, instructional, and assessment methods implemented by accredited Canadian Occupational Therapy programs to capture the perceived impact of these changes on student learning and outcomes.

The baseline study reported increases in program length for certain students due to delays in fieldwork placements and re-sequencing of fieldwork and academic courses to align with public health guidelines (MacKenzie et al., 2023). While four programs in 2021 and one program in 2022 continued to report some residual delay related to fieldwork placements, the current study findings report an overall recovery of program completion to pre-COVID-19 timelines, with no programs reporting delays in program completion as of 2023.

All programs reported a return to pre-COVID-19 delivery methods. While some online synchronous lectures and laboratories remain, there is a noted decrease in the use of online synchronous lectures compared to 2020–2021. The overall shifts were towards pre-COVID-19 delivery of coursework and fieldwork placements (return to in-person delivery and sequencing of curriculum and fieldwork), but some adaptations (such as online synchronous lectures or labs) were maintained. The demonstrated flexibility in academic and fieldwork curricula delivery might not have been previously imagined in a pre-COVID-19 restricted environment. However, the large shifts could not have happened without structural support, human adaptability, and advances in digital tools. Reasons for adopting and maintaining some adaptations included accessibility and accommodations (instructors, facilitators, and students), pedagogy (flipped classroom), logistics (interprofessional education), and others because it worked well with little impact on learning (online PBL).

Programs also reported a return to in-person delivery of assessments because of strong concerns for academic integrity with virtual assessments (i.e., cheating on tests). North et al. (2023) found similar concerns regarding virtual assessments with undergraduate students. Students reported the circumvention of remote exam proctoring was easier than face-to-face proctoring and admitted to having taken advantage of this more often during remote proctoring.

Reported concern regarding practice skills development remained in both academic and fieldwork curricular aspects when practice placements moved to virtual formats (Peart et al., 2022). The hands-on nature of clinical training has been one of the most difficult aspects to replicate in an online environment. During pandemic restrictions, hands-on content was prioritized and delivered in person by moving the teaching and evaluation of physical skills or complex examinations (i.e., OSCE) in the curriculum to when in-person academic programming could occur (MacKenzie et al., 2023). It was noted there is an emerging academic need to prepare students for the evolving service delivery models in occupational therapy, developing practice skills for both in-person and virtual practice environments. However, academic challenges were found with learning intervention skills online and then transferring them to in-person interactions—highlighting the differences in virtual and in-person training for obtaining and using information. When comparing in-person training to synchronous virtual training in a program aimed at supporting family healthy relationships, Gross et al. (2023) found that while both were effective methods of curriculum delivery, the in-person participants gained significantly more knowledge than participants in the virtual group.

The concern regarding communication skills and student anxiety, raised in the previous survey (MacKenzie et al., 2023), was noted again in the present survey. Programs reported perceived impacts on student attainment of competencies due to format changes (impacts on the development of social/interpersonal skills, communication, building rapport, being comfortable with physical touch, building community, professionalism) and assessment method (mostly related to academic integrity issues with online exams). These perceived impacts were previously reported as potential impacts due to delivery changes, but in this follow-up survey, there is now reporting of in-person observable concerns with demonstrated skill development and fieldwork performance. Recent studies exploring the impact of online learning during COVID-19 found the development of soft skills, including clinical reasoning, judgment, and professional expertise, has indeed been impacted in graduate students (Kamysbayeva et al., 2021) as well as in medical students (Vásquez Estrada et al., 2023).

While programs returned to in-person delivery of coursework and fieldwork, the pandemic provided the opportunity for the addition of digital tools for experiential learning and telehealth for fieldwork (Hoel et al., 2021; Proffitt et al., 2021). Simulation of fieldwork experiences (whether in preparation for or as a replacement for real-life fieldwork) has remained in higher use when compared to pre-COVID-19 curriculum delivery methods (MacKenzie et al., 2023). While some studies explored developing simulations for fieldwork equivalency hours (e.g., Rossiter et al., 2023; Sibbald & MacKenzie, 2023), guided by the use of simulation guidelines (Occupational Therapy Council of Australia Ltd., 2020) further studies are needed to explore the potential and limitations of simulation methods for replicating the nuances of in-person interactions and clinical experiences.

In agreement with Gustafsson (2020) the occupational therapy curriculum and fieldwork changes which occurred within a rapid timeframe and under extreme conditions, should be closely examined prior to permanent adoption in an educational program. The adopted changes during the restriction periods highlighted the various ways didactic content could be delivered to students. Notably, many of these shifts were also congruent with the Universal Design for Learning (UDL) principles. Kilpatrick et al. (2021) examined the implementation of UDL by higher education faculty prior to and during the COVID-19 transition. They noted a greater integration of UDL by higher education faculty post-COVID-19, for example, making course concepts available to students through various sources or providing students with additional choices for demonstrating learning.

Securing enough fieldwork placements for students to complete their required hours continued to be an ongoing challenge for programs. Decreased student placement offerings were attributed to burnout in the clinical community and decreased workforce (Lofsky, 2022). Preceptors may be less likely to take on students who need extra support or early learners due to workload considerations. Some programs are now expanding the use of simulation as part of fieldwork (or to replace early fieldwork hours) as a solution, as has been done by others (Grant et al., 2021; Ozelie et al., 2023; Sibbald & MacKenzie, 2024). While there remains continued concern for recovery of clinical fieldwork placements, there was also an appreciation theme for clinical partners for their continued support “… OTs are very committed and connected … and we have a very robust community of wonderful OTs.”

Finally, the core concern of human cost to instructors, the fieldwork community, and students was central to all comments, regardless of the survey question prompt. During the restrictive time periods, faculty members adapted to new roles and responsibilities, including becoming proficient in digital pedagogies. The rapid transition to online education required educators to rethink engagement strategies and explore ways to foster a sense of community and support among students in a virtual environment. This led to an increased focus on professional development opportunities related to online teaching and learning. However, the increased workload during that time period to ensure students completed their programs within the same timeline as pre-COVID-19, left faculty and preceptors in a fatigued or burned-out state in line with other health training programs (O'Brien et al., 2023).

Mental health concerns remained a central issue in the comments of this study. During the restricted COVID-19 time period, there was particular emphasis on attributing social isolation to negative impacts on mental health, interpersonal skills, and potential skill development for practice (MacKenzie et al., 2023). While some students thrived in an online, flexible environment, others struggled with the format and social isolation (Brown et al., 2022). While the impact of social isolation during COVID-19 restrictions was documented in health education programs (Kusumoto et al., 2022), other factors may also have contributed to the vulnerability of being socially isolated. Specifically, other factors such as stress and sleep (Benham, 2021) and generational exposure to technological advances predisposed learners to social isolation prior to the restricted periods of COVID-19 and interaction with modified curricula. There is well-documented evidence that social isolation and loneliness impact mental health (O’Sullivan et al., 2021).

There are several limitations of this study. There is potential for recall bias in providing accurate reporting of data from the academic years of interest. The findings, whether positive or negative, may not apply to all Canadian programs of occupational therapy or regions. While it is important to gather the perceptions of the impact on learner competency from those evaluating students, without concrete program evaluation data to assess, it may not capture the broader community feedback from fieldwork or the student community. Future study is warranted for comparing educational methods related to occupational therapy competency attainment.

## Conclusion

As the field of occupational therapy continues to adapt to the post-COVID-19 era, it is clear that the educational landscape has been indelibly transformed, with implications for the preparedness and skill sets of future practitioners in a world that has been irrevocably changed by the pandemic. As occupational therapy educational programs move forward in a post-pandemic era, it is imperative that the methods used are documented and studied to ensure graduates are well-equipped to meet the evolving needs of the populations they serve.

Through comparative studies, educators and researchers can identify what aspects of the traditional curriculum have been successfully translated to the online environment, what has been lost in the transition, and what new opportunities have emerged. Findings from this study will serve as a foundation from which to track pedagogical changes and their impact on the occupational therapy profession in Canada.

## Key Messages

- The rapid and adaptable curricula shifts during pandemic programming came with human costs—still experienced even after programs returned to pre-pandemic routines.
- Many curriculum changes adopted during the restriction periods were congruent with the Universal Design for Learning (UDL) principles.
- The perceived impact of the online environment on student soft-skill competencies (social/interpersonal skills, communication, rapport building, and professionalism) continues to be a reported concern.
